# Man of Mystery: A Case Report of Dissociative Amnesia in Schizophrenia

**DOI:** 10.7759/cureus.20688

**Published:** 2021-12-25

**Authors:** Keqing Li, William T Yang, Alexander G Perez

**Affiliations:** 1 Psychiatry, St. George's University School of Medicine, Grenada, GRD; 2 Crisis Stabilization Unit (CSU), Jackson Memorial Hospital, Opa Locka, USA

**Keywords:** cognitive impairment and dementia, treatment resistant, schizophrenia and other psychotic disorders, schizophrenia, dissociative amnesia

## Abstract

This case report is unique in its rare presentation of dissociative amnesia with schizophrenia and CT presentation of involutionary prefrontal cortex change. In this case, the patient lost all autobiographical information and memories of his past. He mysteriously appeared in the public health system six months ago without a previous public record and with an alias. In addition, he presented with disorganized behavior, dissociative amnesia, and was internally preoccupied. We diagnosed the patient with schizophrenia and started pharmacological intervention. Unfortunately, the patient had no improvement of insight, and his memory problem persisted despite the treatment. Thus, this case brings attention to dissociative amnesia with comorbid schizophrenia. Moreover, the patient had improvement in mood, but his dissociative amnesia persisted despite medication.

## Introduction

In the Diagnostic and Statistical Manual of Mental Disorders, 5th Edition (DSM-V), dissociative amnesia is the inability to recall important autobiographical information that one should store in memory and would readily remember. It was depicted in popular culture as early as during the Shakespeare era. In this case, we identified generalized amnesia in the patient due to the complete loss of personal identity that’s not pertinent to a specific period [1]. Dissociative amnesia is often related to extreme emotional stress or conflict and is more common among combat veterans and sexual assault victims.

Dissociative amnesia is a rare presentation with a prevalence of 0.2% [2], and very few case reports have been written about it [3-4]. Moreover, the demographics of the disease are more commonly seen among restrictive cultures and females [1]. Very rarely is dissociative amnesia seen presented along with comorbid schizophrenia [5]. Nevertheless, this case demonstrates that dissociative amnesia can co-present with schizophrenia.

## Case presentation

We present a 28-year-old homeless Caucasian man brought in involuntarily by the police for being naked and acting disoriented in public. The medicine ward first admitted the patient for an altered mental status workup and then medically cleared and transferred him to the inpatient psychiatry unit. Later on, the patient was sent to mental health court and was granted six months of inpatient treatment at the state hospital due to self negligence. The patient presented with dissociative amnesia with medical clearance from the ER during this psychiatric hospitalization episode. He was later assessed by inpatient psychiatrists determining that he could not recall any autobiographical information before his first visit to the Jackson health system six months ago despite being oriented to the current person, place, time, and event. 

The patient first appeared in the Jackson health system in April 2021, for which he presented for treatment of cellulitis of his right hand. A few months later, the patient was hospitalized and admitted to the psych ward for altered mental status one time before his current psychiatric hospitalization episode. In this episode, he had acute kidney injury secondary to rhabdomyolysis with a CPK of 1439. In addition, the patient was found to have diabetes mellitus type II and had glucose in the 140 - 200 mg/dL range. The patient was a poor historian and unable to provide any pertinent social history through his past admissions and during this current admission. When asked about his family, the patient repeatedly answered, “I don’t think I have a family.” When we further questioned the question about his childhood and schooling, he replied, “who remembers things in the past? I only live in the moment.” He could not recall any information about his childhood, family, or living area. Later on, he denied having any biological family and stated that “Virgin Mary and Jesus Christ are my mother and father.” The patient admitted to being homeless. Based on our assessment, he did not have any insight into his psychiatric or medical conditions. Interestingly, he tended to get more guarded and agitated whenever examiners questioned about his past. In addition, despite staff explaining to him multiple times what Diabetes is and that he needs insulin for treatment, he remained unable to understand his medical condition and related intervention. The patient was alert and oriented to time, place, and person during his entire hospitalization. In addition, he had no criminal record, and his claimed name is likely an alias due to its unusual sound.

On examination, the patient is a 26-year-old pleasantly psychotic Caucasian man with a tall, medium build who appeared to be in his 50s, poorly groomed, and dressed in a hospital gown. He had good eye contact, cheerful facial expression, and was alert and attentive to the examiner. Moreover, his attitude was intermittently cooperative, guarded, and withdrawn. In addition, he had situational psychomotor agitation. His speech had intermittent pauses, and he could not answer questions intermittently. In addition, his speech was stuttering in rhythm, soft in volume, over-inclusive in amount, dysarthric, and lacking spontaneity. Nevertheless, he consistently reported feeling good. The patient’s affect was stable, full in range, inappropriate content, non-exaggerated in intensity, and euphoric. His thought process was disorganized, incoherent, illogical, loose in the stream, and preserved. He denied suicidal or homicidal ideations and auditory or visual hallucinations but appeared internally preoccupied. He scored 23/30 on Mini-Mental State Examination (MMSE). Specifically, he failed tasks including counting backward from 100 by sevens, spelling WORLD backward, following folding paper instructions, and making up and writing a sentence about anything. 

The patient was diagnosed with schizophrenia and managed on haloperidol 10 mg BID and benztropine 0.5 mg BID for his schizophrenia. In addition, we treated him medically with folic acid 1 mg qd, insulin glargine 100 unit SC qd, sliding scale insulin, metformin 500 mg qhs, multivitamin qd, thiamine HCL 100 mg qd, rosuvastatin 5 mg qd. The patient had improved mood symptoms during his hospitalization but no recollections of any memory. His blood glucose level was consistently below 140 mg/dL.

**Figure 1:**
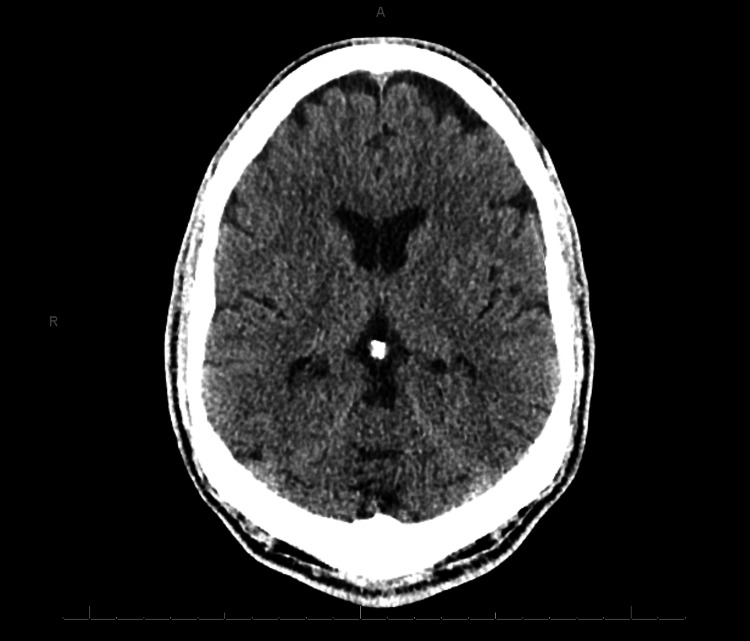
CT-head non-contrast of the patient Radiology described involutionary changes over the prefrontal cortex.

Medically, his blood work was unremarkable besides the initial presentation of elevated CPK and high glucose. His urinalysis had trace ketone and protein of 10. He remained hemodynamically stable. The medical ward did CT secondary to altered mental status characterized by mixed delirium on initial ER assessment, and radiology noted an involuntionary change over the patient’s prefrontal cortex. His CT scan is presented in Figure 1. 

## Discussion

The patient, in this case, suffers from generalized dissociative amnesia resulting from unknown causes. Other differential diagnoses include dissociative identity disorder, posttraumatic stress disorder, and substance-related disorder, which were ruled out by the treatment team due to the absence of pervasive discontinuity in the sense of self and agency, prolonged extension beyond the possible immediate time of trauma, and negative substance screening results. Post-traumatic amnesia due to brain injury is another possible diagnosis, but the lack of general disorientation and confusion and lack of observable head trauma makes it less likely. His amnesia was most prominent in gaps in remote memory, which may be related to dissociative identity disorder [6]. Notably, the patient did appear to be more irritable than his baseline whenever the examiner challenged him about his past. In comparison, the patient did not get agitated when questioned about his medical problems, which he repeatedly denied despite medical advice. His pattern of agitation may indicate severe trauma that may have happened to him, yet he repressed unconsciously, which is typically associated with the dissociative fugue [1]. We saw a similar pattern of agitation in a case report where an Ethiopian woman lost her memory after an ethnic massacre [5]. The patient would also report feeling tense intermittently, possibly indicating his past traumatic experiences resurfacing. Nevertheless, the patient preserved his social skills with his amicable repertoire with examiners and peers. On one occasion, the patient even encouraged the examiner to breathe with him when the examiner mentioned that he felt uneasy at the time.

In addition to the patient’s complete loss of episodic memory, he appeared to have severe insight impairment. He manifested his poor insight as an inability to learn about his medical condition of diabetes and related management. Despite repeated explanations, he continued to understand schizophrenia and diabetes during this episode poorly. This poor insight may be explained as part of his schizophrenia, as this condition tends to have long-lasting inhibition of long-term potentiations related to learning [7-8].

Despite the patient being a poor historian, we gathered two critical pieces of objective data. One is his MMSE score of 23/30, and the other is his CT-head w/o contrast. So far, both parts of the information align with his diagnosis of schizophrenia. Historically, physicians described schizophrenia as dementia praecox [9], a condition presented as early dementia seen paradoxically among young patients. The presentation of cognitive symptoms of schizophrenia also corresponds to this patient’s MMSE. The patient could not solve complex problems despite having the intact capability of simple problem solving and recall.

His loss of complex problem-solving skills also potentially corresponds to the loss of his frontal lobe volume. Secondly, the CT showed involutionary changes over the patient’s prefrontal cortex not seen in other parts of the brain. We may explain the prefrontal cortex loss by the established connection with anatomical changes such as ventricular enlargement and gray matter loss [10-12]. Clinicians often observed prefrontal cortex loss in schizophrenia [13-15]. Involutionary changes can also be seen in the aging brain [16], relating to the appropriate historical reference of dementia praecox.

This case’s limitation is the lack of temporal relationship among prefrontal cortex loss, dissociative amnesia, and schizophrenia. It is unclear whether one presentation was causative to the others or vice versa. In addition, other pharmacotherapies and psychotherapy were not available during this hospitalization due to his lack of mood symptoms, unestablished treatment guidelines, and limitations of hospital resources.

We hope to use this case for establishing efficacious clinical guidelines for treating dissociative amnesia with comorbid schizophrenia. Current experimental treatments for dissociative amnesia include selective serotonin reuptake inhibitor (SSRI) and tricyclic antidepressant (TCA) for mood, trauma-focused psychotherapy for memory retrieval, and neuropsychological rehabilitation for chronic management [17]. ECT in one case has been reported to be successful, the other one precipitating [18-19]. In one similar case where the patient presented with dissociative amnesia and schizophrenia, hypnosis and risperidone were used to improve hostility over the 20 weeks with no resolution of memory loss [5]. Our case study learned that medication and inpatient hospitalization could improve patients’ moods. However, based on this case study and literature review, clinicians should be aware that dissociative amnesia is more treatment-resistant.

## Conclusions

This patient has comorbid schizophrenia and dissociative amnesia, which is a rare case presentation. We hope to provide an example for the management of schizophrenia with dissociative amnesia using our case report. Moreover, a patient's mood can improve with medication, but positive outcomes are less likely for memory recall. Thus, future studies need to establish a clinical guideline for dissociative amnesia with comorbid schizophrenia.
